# Ileus associated with intestinal tuberculosis after initiation of anti-tuberculosis therapy: a case report and scoping review of published cases

**DOI:** 10.1186/s12879-026-14112-w

**Published:** 2026-08-04

**Authors:** Csongor Galko, Zsombor Matics, Edit Hidvegi, Gergely Racz, Veronika Müller

**Affiliations:** 1 Department of Pulmonology, Semmelweis University, Budapest, Hungary; 2 Department of Pathology and Experimental Cancer Research, Semmelweis University, Budapest, Hungary; 3 Department of Pulmonology, Faculty of Medicine, Semmelweis University, Tömő utca 25-29, Budapest, 1083 Hungary

**Keywords:** Ileus, Intestinal tuberculosis, Gastrointestinal obstruction, Anti-tuberculosis therapy, Tuberculosis, Extrapulmonary, Bowel obstruction, Emergency surgery, Case review

## Abstract

**Introduction:**

Tuberculosis remains a significant global health problem, with extrapulmonary involvement occurring in approximately 15–20% of cases. Intestinal tuberculosis may lead to serious complications such as ileus; however, due to the rarity of this condition, the clinical characteristics and outcomes of ileus developing during anti-tuberculosis therapy (ATT) (isoniazid, rifampicin, ethambutol and pyrazinamide) remain poorly defined.

**Objectives:**

To describe the clinical characteristics, timing, management, and outcomes of reported cases of ileus developing after initiation of ATT for intestinal tuberculosis through a scoping review of published literature.

**Methods:**

A scoping review was conducted in accordance with the PRISMA-ScR guidelines. A systematic search of PubMed and Scopus was performed to identify case reports describing intestinal tuberculosis associated with ileus during ATT, covering publications from January 1948 to March 2025. Studies meeting predefined eligibility criteria were screened independently by two reviewers, and relevant clinical data were summarized descriptively. Ileus was categorized as mechanical obstruction (due to stricture, adhesion, inflammatory mass, or wall thickening with a defined transition point) or paralytic ileus (functional impairment without a mechanical obstruction). Additionally, a fatal case from our department was included in the analysis.

**Results:**

A total of 301 records were identified, of which 11 cases met the inclusion criteria. Among these 11 cases, the mean patient age was 49.5 ± 20.0 years; 6/11 were male. Most reported cases originated from Asia, followed by Europe, while one case was reported from Africa. Symptoms typically developed within the first month after initiation of therapy. Abdominal pain was the most frequently reported symptom, occurring in 7/11 cases, followed by vomiting and fever 4/11 cases each. Intestinal obstruction was predominantly mechanical (9/11), and most patients required surgical intervention. One patient received conservative management, and the case from our institution was diagnosed postmortem. Immunosuppressive conditions were documented in 2/11 patients.

**Conclusion:**

Clinicians should maintain a high vigilance for intestinal obstruction in patients with tuberculosis who develop new abdominal pain, vomiting and fever during the first month of ATT as timely diagnostic evaluation is critical. Abdominal computed tomography (CT) imaging and surgical consultation may aid clinical management, although evidence remains limited.

**Clinical trial number:**

Not applicable.

**Supplementary Information:**

The online version contains supplementary material available at https://doi.org/10.1186/s12879-026-14112-w.

## Introduction

Tuberculosis (TB) remains a major global health problem despite advances in diagnosis and treatment. According to the World Health Organization, an estimated 10 million people develop TB annually, and the disease continues to cause significant morbidity and mortality worldwide. The burden of TB is unevenly distributed, with most cases occurring in low- and middle-income countries, particularly in regions of Asia and Africa [1].

International data indicate that approximately 15–20% of tuberculosis patients experience extrapulmonary manifestations, affecting organs such as lymph nodes, pleura, bones, the central nervous system, and the gastrointestinal (GI) tract [1]. Notably, abdominal tuberculosis represents an important subset of extrapulmonary TB and may involve the gastrointestinal tract, peritoneum, lymph nodes, liver or kidneys. Among these forms, intestinal tuberculosis–most commonly affecting the ileocecal region–is one of the most frequently reported presentations [2].

Intestinal tuberculosis is estimated to affect 1–3% of all tuberculosis cases [3]. Clinical manifestations are often nonspecific and may include abdominal pain, weight loss, fever, and altered bowel habits, which can lead to diagnostic delays. The disease may resemble other gastrointestinal disorders such as Crohn’s disease or malignancy, making accurate diagnosis challenging [4]. Its prognosis may be poor, particularly when complicated by perforation, bleeding, or intestinal obstruction.

Intestinal obstruction, including both mechanical obstruction and paralytic ileus, is a rare but potentially life-threatening complication of intestinal tuberculosis. It may develop because of intestinal inflammation, fibrotic stricture formation, adhesions, or paradoxical inflammatory reactions following the initiation of anti-tuberculosis therapy (ATT) (isoniazid, rifampicin, ethambutol and pyrazinamide) [2]. In such cases, rapid clinical deterioration may occur, and urgent surgical intervention may be required. Due to its rarity, the available evidence is largely limited to individual case reports, and summarized data on patient characteristics, timing of onset, and outcomes remain scarce.

The epidemiology and clinical presentation of tuberculosis differ considerably between regions with varying socioeconomic conditions and healthcare resources. In high- and middle-income countries, the incidence of TB is relatively low. In Europe, most countries report fewer than 10 tuberculosis cases per 100,000 population, according to surveillance data from the European Centre for Disease Prevention and Control [3]. In Hungary, the incidence of *Mycobacterium tuberculosis* infection is similarly low, with around 300–500 cases registered annually, and extrapulmonary tuberculosis accounting for only about 3% of reported cases in 2024 [5]. In contrast, low- and middle-income countries carry the overwhelming majority of the global TB burden and more frequently encounter advanced disease stages and complications due to delayed diagnosis and limited healthcare resources [1].

Despite the clinical severity of intestinal obstruction associated with tuberculosis, no standardized patient profile has been established, and the clinical characteristics and outcomes of ileus developing during ATT are not well defined. Addressing this knowledge gap is important for improving early recognition and guiding clinical management in patients receiving treatment for intestinal tuberculosis.

To improve characterization of this uncommon complication, we combined a scoping review of published cases with an additional case from our institution. Therefore, the aim of this study was to describe reported cases of ileus developing after initiation of ATT for intestinal tuberculosis through a scoping review of published literature, including one additional case from our institution.

## Methods

This study was conducted as a scoping review combined with a single case report and was reported in accordance with the Preferred Reporting Items for Systematic Reviews and Meta-Analyses extension for Scoping Reviews (PRISMA-ScR) guidelines. The methodological framework proposed by the Joanna Briggs Institute is presented in Supplementary Table 1 [6]. The aim of the scoping review was to identify and summarize published case reports and case series describing intestinal obstruction (ileus) associated with intestinal tuberculosis during ATT.

A comprehensive literature search was performed in the electronic databases PubMed and Scopus to identify published case reports and case series describing ileus associated with intestinal tuberculosis during ATT. The search covered publications from January 1, 1948, to March 31, 2025, and the final search was conducted on March 31, 2025. The same Boolean search strategy was applied in both databases using database-specific syntax: (“tuberculosis” OR tuberculosis OR TB) AND (ileus OR “intestinal obstruction” OR intestinal) AND (“case report” OR “case series”). No language, geographic, publication status, or study-design filters were applied during database searching. Articles published in languages other than English were translated for eligibility assessment and data extraction. To ensure that no additional eligible reports had become available during the revision process, the literature search was repeated on July 20, 2026, using the same search strategy. No additional eligible case reports or case series were identified. In addition, the reference lists of all included articles were manually screened to identify any further eligible studies. Embase was considered during the study design but was not included because of resource limitations. The search was based on free-text Boolean search terms and did not incorporate controlled vocabulary (e.g., MeSH or Emtree terms). Grey literature sources were not searched because the review focused on published peer-reviewed case reports and case series. A total of 301 records were identified through database searching. The complete search strategies are provided in Supplementary Table 2.

Studies were considered eligible for inclusion if they reported human patients diagnosed with intestinal tuberculosis presenting with ileus or intestinal obstruction and were published as case reports or case series with sufficient clinical information relevant to the study objectives. Studies were excluded if they did not involve intestinal tuberculosis, did not report ileus or intestinal obstruction, were review articles, editorials, conference abstracts, or animal studies, lacked sufficient clinical detail for data extraction, or represented duplicate publications. Reasons for exclusion during full-text review were documented and are summarized in the PRISMA-ScR flow diagram.

To ensure consistent case classification, intestinal tuberculosis had to be documented as the direct cause of ileus or intestinal obstruction during ATT. Patients with pulmonary, renal, peritoneal, or other extrapulmonary tuberculosis were included only when intestinal tuberculosis subsequently developed and was identified in the original report as the cause of the obstructive gastrointestinal event. Likewise, reports describing paradoxical inflammatory (IRIS-like) phenomena were included only when intestinal tuberculosis remained the underlying cause of ileus rather than representing an alternative diagnosis.

All identified records were imported into a reference management software program, and duplicate records were removed. The screening process was conducted independently by two reviewers (CG and ZM) in two stages: title and abstract screening followed by full-text review. Any disagreements between reviewers were resolved through discussion, and if consensus could not be reached, a third reviewer (VM) was consulted to make the final decision.

Data from all eligible studies were charted using a standardized data extraction form. The collected variables included age, sex, other organ involvement, symptoms, ileus type, diagnostic confirmation, need of surgery, treatment, onset of ileus, outcome, underlying immunocompromising disease, surgical procedure, and follow up. For the purposes of this review, *mechanical obstruction* was defined as a physical blockage of the intestinal lumen caused by tuberculosis-related stricture, adhesions, inflammatory mass, or bowel wall thickening confirmed by imaging, surgery, or autopsy findings. *Paralytic ileus* was defined as functional impairment of bowel motility without evidence of a fixed mechanical transition point or obstructing lesion. Cases were categorized according to the descriptions provided in the original reports. Five included articles were originally published in Japanese (*n* = 4) and Greek (*n* = 1) and were translated into English language using an artificial intelligence-assisted translation tool (Claude 3.7 Sonnet). The translated text was manually reviewed by the investigators, and the extracted clinical variables (e.g., demographic characteristics, clinical presentation, diagnostic findings, treatment, and outcomes) were cross-checked against the original publication, including tables, figures, and numerical data where applicable, to ensure consistency before data extraction. In accordance with scoping review methodology, the data were summarized descriptively. A methodological quality appraisal of the included case reports was performed using the Joanna Briggs Institute (JBI) Critical Appraisal Checklist for Case Reports. However, no quantitative risk-of-bias synthesis or meta-analysis was undertaken because of the descriptive nature of the review. Missing data were recorded as not reported when relevant variables were unavailable in the original publications, and analyses were conducted using the available data only. No attempts were made to contact the authors of the original reports for additional information.

## Results

**Fig. 1:**
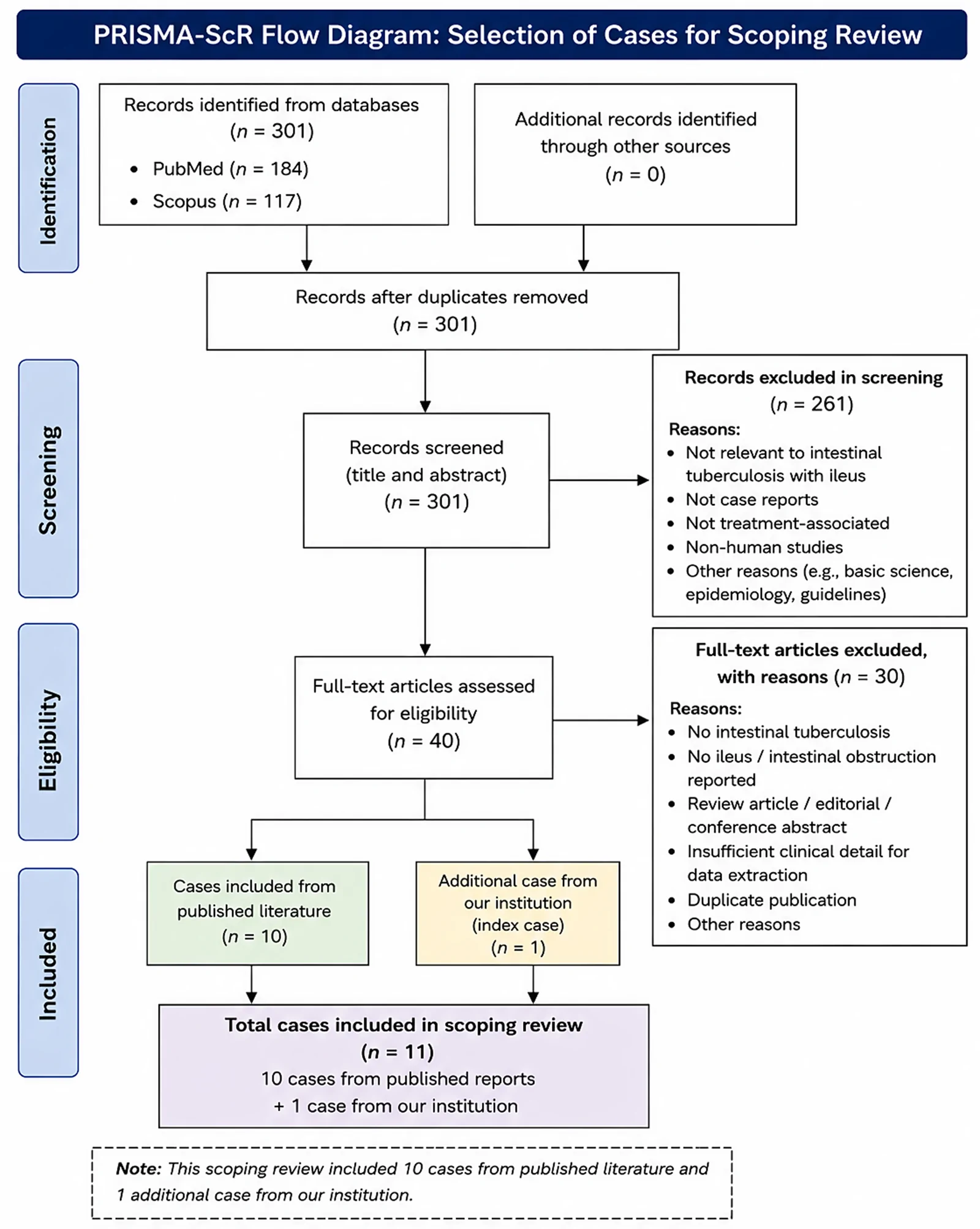
PRISMA-ScR flow diagram illustrating study identification, screening, eligibility assessment, and inclusion process for the scoping review, including reasons for article exclusion during screening and full-text review

The literature search identified 10 published cases of ileus associated with intestinal tuberculosis occurring during ATT that fulfilled the predefined eligibility criteria. In addition, the institutional index case described in this report also fulfilled all eligibility criteria and was included in the overall study dataset, resulting in a total of 11 cases. To improve methodological transparency, findings from the published literature are distinguished from those of the complete dataset, including the institutional case, where appropriate. The study identification, screening, eligibility assessment, and inclusion process are summarized in Fig. 1.

Methodological quality appraisal using the Joanna Briggs Institute (JBI) Critical Appraisal Checklist for Case Reports indicated that most included reports adequately described patient demographics, clinical presentation, diagnostic methods, and interventions, whereas reporting of follow-up and post-intervention outcomes was less consistent.

**Fig. 2:**
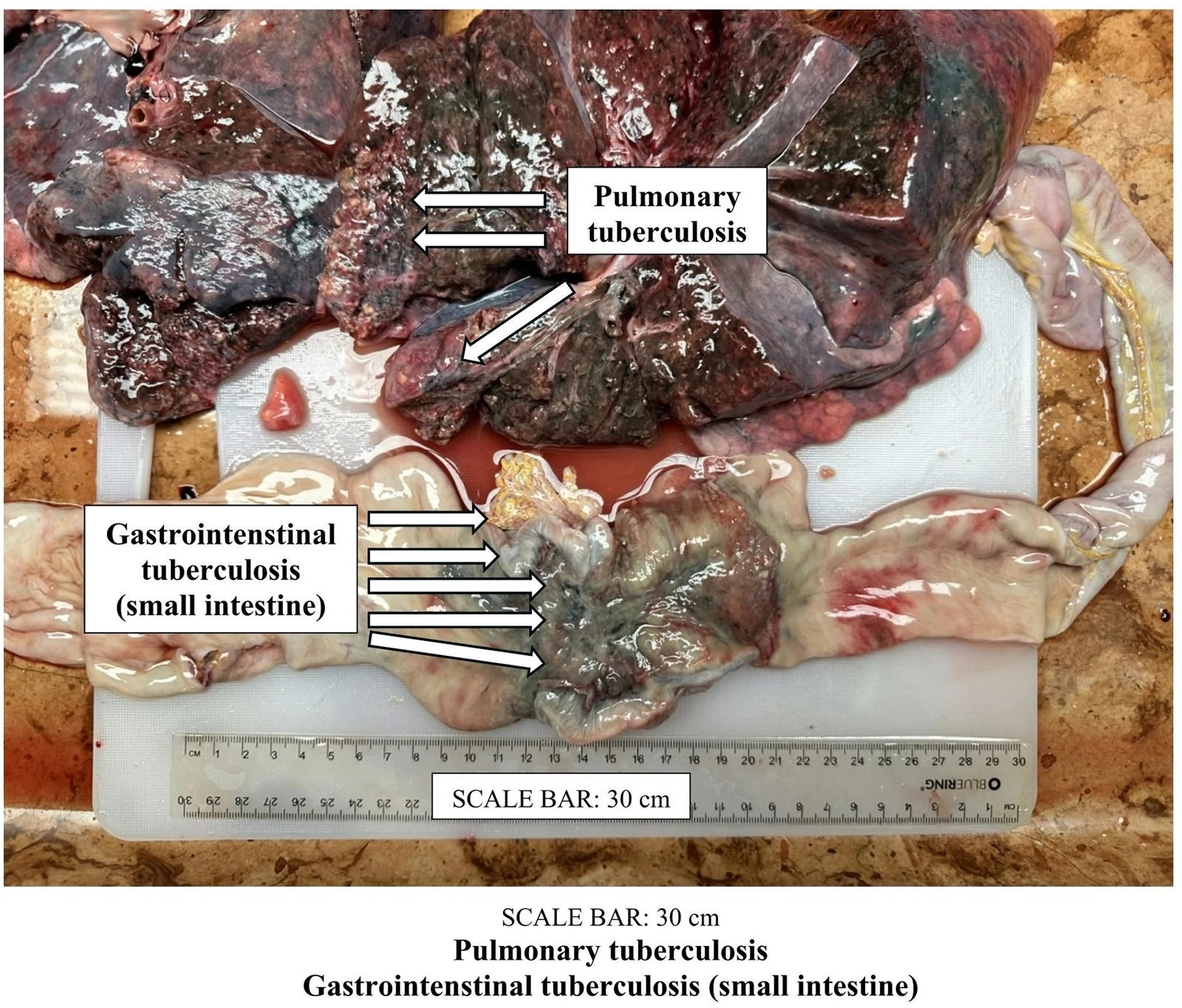
Gross pathological findings at autopsy demonstrating pulmonary tuberculosis with scattered caseating granulomas and ulcerative-hypertrophic intestinal tuberculosis involving the small intestine with transmural inflammatory destruction

**Fig. 3:**
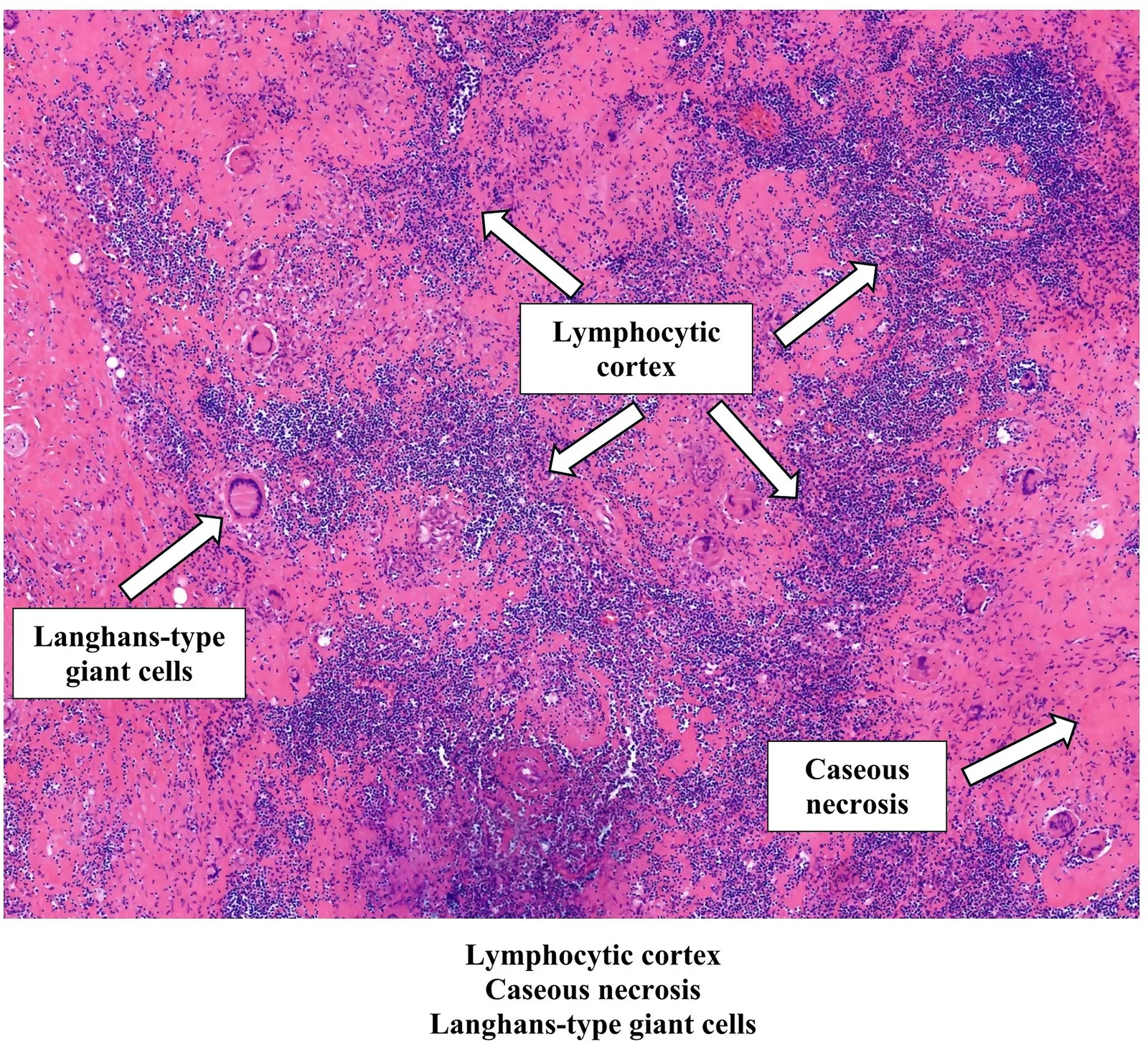
Hematoxylin and eosin (H&E)-stained histopathological section of the small intestine demonstrating caseating granulomatous inflammation with Langhans-type giant cells and lymphocytic infiltrates consistent with intestinal tuberculosis

A 35-year-old male presented with significant weight loss (30 kg over 3 months) and odynophagia. Enlarged cervical lymph nodes and upper-lobe-predominant chest computed tomography findings suggested pulmonary tuberculosis. Acid-fast bacilli were detected in sputum samples, and microbiological testing later confirmed *Mycobacterium tuberculosis* infection. Baseline laboratory investigations demonstrated evidence of systemic inflammation C-reactive protein (CRP 62.1 mg/L), without increase in procalcitonin (0.23 µg/L). Total white blood cell count was 5.10 G/L with neutrophilia (3.82 G/L, 74.9%), lymphocytopenia (0.58 G/L, 11.4%), and microcytic anemia (hemoglobin 92 g/L, mean corpuscular volume 68.3 fL) and normal platelet count (348 G/L). Serum sodium and potassium levels were within the normal range (140 mmol/L and 3.7 mmol/L, respectively). Initiation of standard four-drug ATT (isoniazid, rifampicin, ethambutol and pyrazinamide) resulted in significant clinical improvement, including a 6 kg weight gain during the first 2.5 weeks. However, on the 18th day of treatment, the patient developed sudden-onset abdominal pain, vomiting, and diarrhea, followed within hours by rapidly progressive mechanical ileus and metabolic acidosis consistent with an acute abdominal emergency. Urgent surgical consultation was requested, and emergency abdominal computed tomography imaging was planned for suspected intestinal obstruction. During transfer for imaging, circulatory collapse and respiratory failure occurred. Despite immediate resuscitation efforts, cardiorespiratory failure was irreversible. Autopsy revealed mechanical ileus caused by ulcerative-hypertrophic intestinal tuberculosis localized to the ileocecal region (Fig. 2). Histopathological examination confirmed *Mycobacterium tuberculosis* infection (Fig. 3). According to the patient’s history, intestinal tuberculosis could have originated from the consumption of raw milk from infected cattle or developed as a secondary infection following ingestion of acid-fast bacilli.

Including our index case, 11 cases of ileus associated with intestinal tuberculosis during ATT were identified. The demographic, clinical, diagnostic, and therapeutic characteristics of the included cases are summarized in Table 1. Although several patients had pulmonary, renal, or peritoneal tuberculosis, gastrointestinal tuberculosis was present in all included cases and was considered the direct cause of ileus according to the original reports. Reports originated predominantly from Asia, followed by Europe and Africa, with no cases reported from the American continent. The median patient age was 44 years (range 25–89 years), while the mean age was 49.5 ± 20.0 years. Immunocompromising conditions (HIV infection) were present in 2 of 11 cases.

Although 6 of the 11 cases developed within the first month after treatment initiation, several reports described delayed obstruction occurring after prolonged therapy, suggesting heterogeneous underlying mechanisms. Abdominal pain was the most frequently reported symptom 7 of 11 cases, followed by vomiting and fever 4 cases each, while weight loss was reported in 3 cases and constipation or dysphagia in isolated cases. Progression commonly led to mechanical intestinal obstruction requiring urgent surgical intervention.

Mechanical obstruction was reported in 9 of 11 cases, whereas paralytic ileus was described in 2 of 11 cases. CT imaging was the primary diagnostic modality, commonly demonstrating bowel wall thickening, transition points, strictures, or proximal bowel dilatation consistent with intestinal obstruction. Colonoscopy was used in one case, and one diagnosis was established postmortem at autopsy. Most patients required surgical intervention due to acute clinical deterioration. Laparotomy was the most frequently performed procedure. Overall, surgical management was required in 9/11 cases. One patient was successfully managed conservatively, and one case was diagnosed postmortem due to acute cardiorespiratory collapse. Postoperative follow-up was reported in selected cases, ranging from 4 to 12 months, while follow-up data were unavailable for other reports.

## Discussion

This scoping review identified 11 reported cases of intestinal tuberculosis complicated by ileus occurring during ATT. Although rare, this complication is clinically important due to its potential for rapid deterioration and frequent need for emergency surgical intervention.

Across the included cases, gastrointestinal symptoms most commonly appeared within the first 2–4 weeks after initiation of standard four-drug ATT. The most frequently reported symptoms included abdominal pain (7 cases), vomiting and fever (4 cases each), and weight loss (3 cases). In the majority of cases, clinical progression culminated in mechanical intestinal obstruction requiring urgent surgical management in most reported cases. Abdominal CT was the principal diagnostic modality, often supplemented by intraoperative findings, and remains central for detecting characteristic features of intestinal tuberculosis-associated obstruction, including concentric bowel wall thickening, ileocecal involvement, luminal narrowing with proximal bowel dilatation, inflammatory strictures, mesenteric lymphadenopathy, and adjacent inflammatory fat stranding. In several cases, CT findings also suggested transition points consistent with mechanical obstruction. Early diagnosis of abdominal tuberculosis relies on a combination of clinical suspicion, imaging findings, and microbiological or histopathological confirmation [2, 7, 8].

Most reported cases originated from Asia and the frequent presentation of a symptom triad (abdominal pain, vomiting, and fever) in this region may reflect differences in baseline tuberculosis burden, diagnostic delay, or healthcare access. However, these observations should be interpreted cautiously due to the limited number of cases and potential reporting bias. Notably, only 2 of 11 patients were immunocompromised, suggesting that intestinal tuberculosis leading to ileus may occur in immunocompetent individuals [9].

Differential diagnosis of gastrointestinal deterioration during ATT remains clinically challenging. Intestinal tuberculosis may closely mimic Crohn’s disease because both conditions can present with abdominal pain, weight loss, strictures, ulceration, bowel wall thickening, and ileocecal involvement [4, 7]. In addition, postoperative or inflammatory adhesions, primary gastrointestinal malignancies, and nonspecific infectious or inflammatory enterocolitis may also contribute to bowel obstruction or overlapping radiologic findings. Drug-related gastrointestinal adverse effects may further complicate recognition of evolving intestinal obstruction. These manifestations may include nausea, vomiting, abdominal distension, hepatotoxicity-related symptoms, or reduced bowel motility. Careful integration of clinical presentation, imaging findings, microbiological confirmation, histopathology, and treatment response is therefore essential for accurate diagnosis and timely management [7, 8].

The temporal association between initiation of therapy and symptom onset raises the possibility of a paradoxical inflammatory response, potentially analogous to immune reconstitution inflammatory syndrome (IRIS)-like phenomena. Although IRIS is classically described in HIV-associated tuberculosis, paradoxical worsening after initiation of ATT has also been reported in HIV-negative patients with extrapulmonary and abdominal tuberculosis. These reactions may manifest as enlargement of pre-existing lesions, new inflammatory masses, worsening lymphadenopathy, ascites, bowel wall edema, or development of intestinal strictures despite appropriate microbiological treatment and initial clinical improvement [8, 10]. In abdominal tuberculosis, paradoxical responses have been described particularly during the early weeks of treatment. However, the underlying mechanisms remain incompletely understood and may involve dysregulated inflammatory responses following rapid mycobacterial killing [11].

Enhanced antigen release during effective therapy may trigger exaggerated local inflammation in intestinal lesions. This process may contribute to edema, fibrosis, adhesions, or progression of previously subclinical strictures, ultimately precipitating mechanical intestinal obstruction. However, this mechanism remains speculative in the present review, as none of the included reports provided immunological, histopathological, or biomarker-based confirmation of paradoxical inflammatory syndrome.

The variable timing of intestinal obstruction observed across the included cases may reflect different underlying pathogenic mechanisms. Cases developing within the first weeks after initiation of ATT may be more consistent with acute inflammatory responses, including paradoxical reaction or IRIS-like immune activation. These changes may exacerbate previous subclinical lesions and result in early luminal narrowing or impaired intestinal motility. In contrast, cases presenting later during therapy may reflect progressive fibrosis, cicatrization, adhesion formation, or evolution of chronic strictures secondary to healing intestinal tuberculosis lesions. Mechanical obstruction developing after several months of therapy may represent a delayed structural consequence of chronic inflammation. This presentation may differ from the acute inflammatory pattern observed early after treatment initiation. Paralytic ileus may additionally arise through severe systemic inflammation, electrolyte imbalance, or diffuse intestinal dysmotility without fixed obstruction. Although the limited number of reported cases precludes definitive mechanistic classification, this temporal framework may help explain the heterogeneity in clinical presentation and timing of obstruction during ATT.

The findings of this review are hypothesis-generating rather than predictive. Although based on a limited number of published cases, they suggest that close clinical surveillance during the early phase of ATT may be valuable, particularly within the first month of treatment. New or worsening gastrointestinal symptoms during this period should prompt careful clinical evaluation rather than being attributed solely to adverse effects of ATT. In the published cases, abdominal CT was the most used imaging modality to investigate suspected intestinal obstruction, and surgical consultation was frequently required in patients with clinical deterioration. These observations should be interpreted as descriptive findings rather than evidence that CT imaging or early surgical intervention improves clinical outcomes [6, 7].

Given that most reported patients required surgical intervention, early multidisciplinary involvement, including surgical consultation, may be warranted in cases of suspected ileus. Awareness of this complication remains important in both high- and low-tuberculosis-burden regions. Extrapulmonary manifestations may otherwise be underrecognized or misattributed to alternative gastrointestinal conditions such as malignancy or inflammatory bowel disease [6].

Furthermore, epidemiological data indicate that extrapulmonary tuberculosis accounts for a substantial proportion of tuberculosis cases in Europe, underscoring the need for vigilance in recognizing atypical presentations. Clinical monitoring during the early phase of treatment may facilitate earlier recognition of potential complications; however, the available evidence is insufficient to determine whether this approach improves patient outcomes [1, 3].

This scoping review has several limitations. First, the small number of reported cases limits the ability to draw definitive conclusions. Second, publication bias is likely, as severe or surgically managed cases may be more frequently reported, while milder cases remain underrepresented. Third, incomplete clinical and follow-up data in several reports restricted assessment of long-term outcomes. Fourth, a geographic reporting imbalance, with a predominance of cases from Asia, may limit generalizability to other regions. Notably, all reported Asian cases originated from Japan and Taiwan, despite the higher tuberculosis burden in South/Southeast Asia and Africa. This likely reflects publication bias and differences in healthcare access and reporting rather than a true absence of cases in these regions. Additionally, recognition and screening of gastrointestinal tuberculosis are not standardized, which may contribute to delayed diagnosis and inconsistent reporting. Distinguishing gastrointestinal symptoms related to disease progression from adverse effects of ATT can be challenging in clinical practice. Finally, emergency cases requiring urgent surgical intervention may be underreported due to practical and publication-related constraints. Additionally, incomplete reporting of ATT duration and possible adjunctive corticosteroid use limited further assessment of factors potentially associated with paradoxical inflammatory responses. Furthermore, five non-English publications were translated using an AI assisted translation approach with manual verification by the investigators [12]. Although this approach enabled inclusion of otherwise inaccessible reports, subtle nuances in the original text may have been lost, introducing a potential risk of misclassification or data extraction bias. Finally, the literature search was limited to PubMed and Scopus. Although the reference lists of all included articles were manually screened to identify additional eligible studies, relevant reports indexed exclusively in other databases, such as Embase, may have been missed.

In summary, intestinal tuberculosis-associated ileus during ATT is a rare but clinically important complication. In the published cases reviewed, new or worsening abdominal symptoms most occurred during the early phase of treatment and were frequently evaluated with abdominal CT, with many patients subsequently undergoing surgical intervention. These findings should be regarded as clinical observations derived from a small number of case reports rather than evidence-based recommendations. Further prospective and mechanistic studies are needed to clarify the pathogenesis of this complication and to determine optimal diagnostic and therapeutic strategies.

**Table 1:** Patient demographics and symptoms

Patient number	Country of reporting	Age	Sex	Extraintestinal TB involvement	Symptoms	Ileus type	Diagnostic Confirmation
1.	Hungary	35	Male	Lung	Weight loss, Odynophagia	Mechanical	Autopsy
2.	Japan [13]	44	Male	Lung and Kidney	Acute abdominal pain, Intermittent fever	Paralytic	CT
3.	Japan [14]	44	Male	Lung	Abdominal pain, Vomiting	Mechanical	CT, surgery
4.	Japan [15]	25	Female	Intestines	Right lower abdominal pain, Fever	Mechanical	CT, surgery
5.	Taiwan [16]	44	Male	Lung	Abdominal distension and dull pain, Vomiting	Paralytic	CT, surgery
6.	Germany [17]	52	Female	Lung and GI	Abdominal pain	Mechanical	CT, surgery
7.	Switzerland [18]	30	Male	Ileocecal	Acute abdominal pain, Fever	Mechanical	Surgery
8.	South Africa [19]	35	Female	Lung and Colon	Intermittent abdominal pain, Vomiting, Long-standing constipation, Loss of weight, Abdominal distension	Mechanical	CT, surgery
9.	Japan [20]	89	Female	Peritoneum	Vomiting, Fever	Mechanical	CT
10.	Japan [21]	73	Female	Ileocecal	Abdominal pain, Weight loss	Mechanical	CT, colonoscopy
11.	Greece [22]	68	Male	Lung	No Data	Mechanical	CT
	**Need of surgery**	**Treatment**	**Time from ATT initiation to ileus**	**Outcome**	**Underlying immunocompromising disease**	**Surgical Procedure**	**Follow up**
1.	No	ATT	≤ 30 days	Death	No	None	Not reported
2.	No	ATT	≤ 30 days	Successful conservative care	No	None	Not reported
3.	Yes	ATT	≤ 30 days	Successful treatment	No	Laparotomy	6 months
4.	Yes	ATT	≤ 30 days	Successful treatment	No	Laparotomy	Not reported
5.	Yes	ATT	31–90 days	Successful treatment	No	Laparotomy	Not reported
6.	Yes	ATT	≤ 30 days	Successful treatment	No	Laparotomy	12 months
7.	Yes	ATT	> 90 days	Successful treatment	HIV	Laparotomy	Not reported
8.	Yes	ATT	> 90 days	Successful treatment	HIV	Laparotomy	8 months
9.	Yes	ATT	31–90 days	Successful treatment	No	Laparotomy	4 months
10.	Yes	ATT	≤ 30 days	Successful treatment	No	Laparotomy	Not reported
11.	Yes	ATT	No Data	Successful treatment	No	Surgery	Not reported

GI= gastrointestinal

CT= computed tomography

ATT= anti-tuberculosis treatment (Isoniazid (INH), Rifampin (RIF), Pyrazinamide (PZA), and Ethambutol (EMB))

HIV= human immunodeficiency virus

## Supplementary Information

Below is the link to the electronic supplementary material.

Supplementary Material 1

Supplementary Material 2

## Data Availability

No datasets were generated or analysed during the current study.

## Abbreviations

TB: Tuberculosis
CT: Computed Tomography
M. tuberculosis: Mycobacterium tuberculosis
SD: Standard Deviation
HIV: Human Immunodeficiency Virus
WHO: World Health Organization
ECDC: European Centre for Disease Prevention and Control
ATT: Anti-tuberculosis Therapy
IRIS: Immune Reconstitution Inflammatory Syndrome
