# Supplementary material for: Ileus associated with intestinal tuberculosis after initiation of anti-tuberculosis therapy: a case report and scoping review of published cases
Source: BMC Infect Dis. 2026 Aug 4;26:1920. doi: 10.1186/s12879-026-14112-w (PMC13726356; doi:10.1186/s12879-026-14112-w)
Supplement: Supplementary file 1 [file 12879_2026_14112_MOESM1_ESM.pdf]

Supplementary Table 2. Database-specific search strategies used for the scoping review

Database	Search strategy	Date searched	Filters applied	Language restrictions
PubMed	("tuberculosis" OR tuberculosis OR TB) AND (ileus OR "intestinal obstruction" OR intestinal) AND ("case report" OR "case series")	31 March 2025	None	None
Scopus	("tuberculosis" OR tuberculosis OR TB) AND (ileus OR "intestinal obstruction" OR intestinal) AND ("case report" OR "case series")	31 March 2025	None	None

Additional search details

- Search period: January 1, 1948 – March 31, 2025
- Databases searched: PubMed and Scopus
- Final search date: March 31, 2025
- Language restrictions: None. Articles published in languages other than English were translated for eligibility assessment and data extraction.
- Publication restrictions: None.
- Geographic restrictions: None.
- Manual searching: Reference lists of all included articles were screened to identify additional eligible studies.
- Additional databases: Embase was considered but was not searched because institutional access was unavailable.
- Total records identified through database searching: 301.
