# Supplementary material for: Ileus associated with intestinal tuberculosis after initiation of anti-tuberculosis therapy: a case report and scoping review of published cases
Source: BMC Infect Dis. 2026 Aug 4;26:1920. doi: 10.1186/s12879-026-14112-w (PMC13726356; doi:10.1186/s12879-026-14112-w)
Supplement: Supplementary file 2 [file 12879_2026_14112_MOESM2_ESM.pdf]

JBI checklist

Case	First author	Patient demographics described	History described	Clinical presentation described	Diagnostics described	Intervention described	Outcome described	Adverse events described	Takeaway lesson reported	JBI score
1.	Our case	Yes	Yes	Yes	Yes	Yes	Yes	Yes	Yes	8/8
2.	Hashiba T.	Yes	Yes	Yes	Yes	Yes	Yes	Yes	Yes	8/8
3.	Ida N.	Yes	Yes	Yes	Yes	Yes	Yes	Yes	Yes	8/8
4.	Miura T.	Yes	Yes	Yes	Yes	Yes	Yes	Yes	Yes	8/8
5.	Lee CW.	Yes	Yes	Yes	Yes	Yes	Yes	Yes	Yes	8/8
6.	Wurnig PN.	Yes	Yes	Yes	Yes	Yes	Yes	Yes	Yes	8/8
7.	Guex AC.	Yes	Yes	Yes	Yes	Yes	Yes	Yes	Yes	8/8
8.	Steer D.	Yes	Yes	Yes	Yes	Yes	Yes	Yes	Yes	8/8
9.	Takeuchi N.	Yes	Yes	Yes	Yes	Yes	Yes	Yes	Yes	8/8
10.	Matsumoto Yumi	Yes	Yes	Yes	Yes	Yes	Yes	Yes	Yes	8/8
11.	Drivala M.	Yes	Yes	Yes	Yes	Yes	Yes	Yes	Yes	8/8
